# An inline deep learning based free-breathing ECG-free cine for exercise cardiovascular magnetic resonance

**DOI:** 10.1186/s12968-022-00879-9

**Published:** 2022-08-11

**Authors:** Manuel A. Morales, Salah Assana, Xiaoying Cai, Kelvin Chow, Hassan Haji-valizadeh, Eiryu Sai, Connie Tsao, Jason Matos, Jennifer Rodriguez, Sophie Berg, Neal Whitehead, Patrick Pierce, Beth Goddu, Warren J. Manning, Reza Nezafat

**Affiliations:** 1 Department of Medicine (Cardiovascular Division), Beth Israel Deaconess Medical Center and Harvard Medical School, 330 Brookline Ave., Boston, MA 02215 USA; 2 Siemens Medical Solutions USA, Inc, Chicago, IL USA; 3 Department of Radiology, Beth Israel Deaconess Medical Center and Harvard Medical School, Boston, MA USA

**Keywords:** Coronary artery disease, Exercise MRI, Deep learning, Radial golden angle, Inline

## Abstract

**Background:**

Exercise cardiovascular magnetic resonance (Ex-CMR) is a promising stress imaging test for coronary artery disease (CAD). However, Ex-CMR requires accelerated imaging techniques that result in significant aliasing artifacts. Our goal was to develop and evaluate a free-breathing and electrocardiogram (ECG)-free real-time cine with deep learning (DL)-based radial acceleration for Ex-CMR.

**Methods:**

A 3D (2D + time) convolutional neural network was implemented to suppress artifacts from aliased radial cine images. The network was trained using synthetic real-time radial cine images simulated using breath-hold, ECG-gated segmented Cartesian k-space data acquired at 3 T from 503 patients at rest. A prototype real-time radial sequence with acceleration rate = 12 was used to collect images with inline DL reconstruction. Performance was evaluated in 8 healthy subjects in whom only rest images were collected. Subsequently, 14 subjects (6 healthy and 8 patients with suspected CAD) were prospectively recruited for an Ex-CMR to evaluate image quality. At rest (*n* = 22), standard breath-hold ECG-gated Cartesian segmented cine and free-breathing ECG-free real-time radial cine images were acquired. During post-exercise stress (*n* = 14), only real-time radial cine images were acquired. Three readers evaluated residual artifact level in all collected images on a 4-point Likert scale (1-non-diagnostic, 2-severe, 3-moderate, 4-minimal).

**Results:**

The DL model substantially suppressed artifacts in real-time radial cine images acquired at rest and during post-exercise stress. In real-time images at rest, 89.4% of scores were moderate to minimal. The mean score was 3.3 ± 0.7, representing increased (P < 0.001) artifacts compared to standard cine (3.9 ± 0.3). In real-time images during post-exercise stress, 84.6% of scores were moderate to minimal, and the mean artifact level score was 3.1 ± 0.6. Comparison of left-ventricular (LV) measures derived from standard and real-time cine at rest showed differences in LV end-diastolic volume (3.0 mL [− 11.7, 17.8], P = 0.320) that were not significantly different from zero. Differences in measures of LV end-systolic volume (7.0 mL [− 1.3, 15.3], P < 0.001) and LV ejection fraction (− 5.0% [− 11.1, 1.0], P < 0.001) were significant. Total inline reconstruction time of real-time radial images was 16.6 ms per frame.

**Conclusions:**

Our proof-of-concept study demonstrated the feasibility of inline real-time cine with DL-based radial acceleration for Ex-CMR.

**Supplementary Information:**

The online version contains supplementary material available at 10.1186/s12968-022-00879-9.

## Background

Stress cardiac imaging is the first-line provocative test for the diagnosis and prognosis of coronary artery disease (CAD). During the past decade, stress cardiovascular magnetic resonance (CMR) imaging has become widely used clinically, and is increasingly recognized as an important testing tool in CAD [1–5]. CMR has many well established benefits over other imaging modalities such as fewer limitations from body habitus and no ionizing radiation. CMR cine imaging is also the non-invasive gold standard for assessment of left-ventricular (LV) anatomy and function and may be utilized for detection of stress-induced LV regional wall motion abnormalities [6, 7]. Nevertheless, current clinical CMR requires pharmacological stimulation to induce stress, which does not elicit the same cardiovascular response as physical stress [8–10] and lacks important prognostic data [11, 12].

Coalescing technical innovations in CMR over the past 25 years are enabling CMR imaging during exercise (Ex-CMR) as an alternative to pharmacologic stress, including the advent of highly accelerated real-time cine sequences [13]. Ex-CMR was initially demonstrated by Weiss et al. using isometric handgrip exercise [14]. Later studies using a CMR-compatible bicycle ergometer required breath-holding to evaluate cardiac function during exercise stress [15, 16], which was problematic as breath-holds during exercise are difficult to perform. Lurz et al. later proposed a radial *k-t* SENSE sequence to assess biventricular volume and function during free-breathing continuous exercise [17]. However, an electrocardiogram (ECG) signal was needed to retrospectively reconstruct images, and ECG gating issues during maximal exercise intensity compromised image quality [18, 19]. Thus, a number of clinical studies since then have relied on the ungated real-time sequence proposed by La Gerche et al. using Cartesian k-space undersampling [20]. More recently, Haji-valizadeh et al. reported a free-breathing ECG-free real-time radial cine sequence with higher spatiotemporal resolution [21] using the golden-angle radial sparse parallel (GRASP) reconstruction framework [22]. Nevertheless, without specialized techniques such as parallel imaging and compressed sensing, highly accelerated cine images suffer from significant aliasing artifacts that can degrade LV assessment or render images non-diagnostic. From a clinical perspective, a practical yet crucial limitation of these specialized strategies is that reconstruction times can be long and not truly real-time. Alternatively, faster image reconstruction and de-aliasing of Cartesian [23–25] and radial [26–28] data is feasible with deep learning (DL). However, most methods have been evaluated on retrospectively undersampled or breath-hold images. Further, DL is yet to be evaluated for Ex-CMR, which is potentially more difficult since real-time images acquired during stress may have increased artifacts compared to rest.

In this study, we sought to develop an inline (i.e., on-scanner) DL-based approach for seamless, low-latency image acquisition and reconstruction of real-time ECG-free radial cine with an acceleration rate of 12 for Ex-CMR. In contrast to previous methods that use magnitude images for learning, our model was trained using complex-valued images simulated from raw k-space data acquired at rest. This approach preserves phase information in the images. We hypothesized that a DL model trained for image de-aliasing with phase-preserved, at-rest images is robust to the significant respiratory and cardiac motion that occurs during exercise stress. We evaluated and compared our method to GRASP on healthy subjects and patients with suspected CAD referred to Ex-CMR.

## Methods

A DL radial acceleration with parallel reconstruction (DRAPR) model based on a three-dimensional U-Net was implemented to suppress streaking artifacts in highly accelerated real-time radial cine images. The model was trained using real-time radial images synthesized from conventional ECG-gated segmented Cartesian k-space data from 503 patients at rest. An inline pipeline was then implemented to enable fast, parallel reconstruction during a prospective Ex-CMR study.

### Population

The study protocol was approved by the Beth Israel Deaconess Medical Center Institutional Review Board (IRB). Two cohorts were included in this study. For the training cohort, the IRB waived written informed consent to retrospectively analyze CMR data. For the prospective validation cohort, written informed consent was obtained from each participant. For both cohorts, patient information was handled in compliance with the Health Insurance Portability and Accountability Act.

### Deep learning model

#### Network architecture

**Fig. 1:**
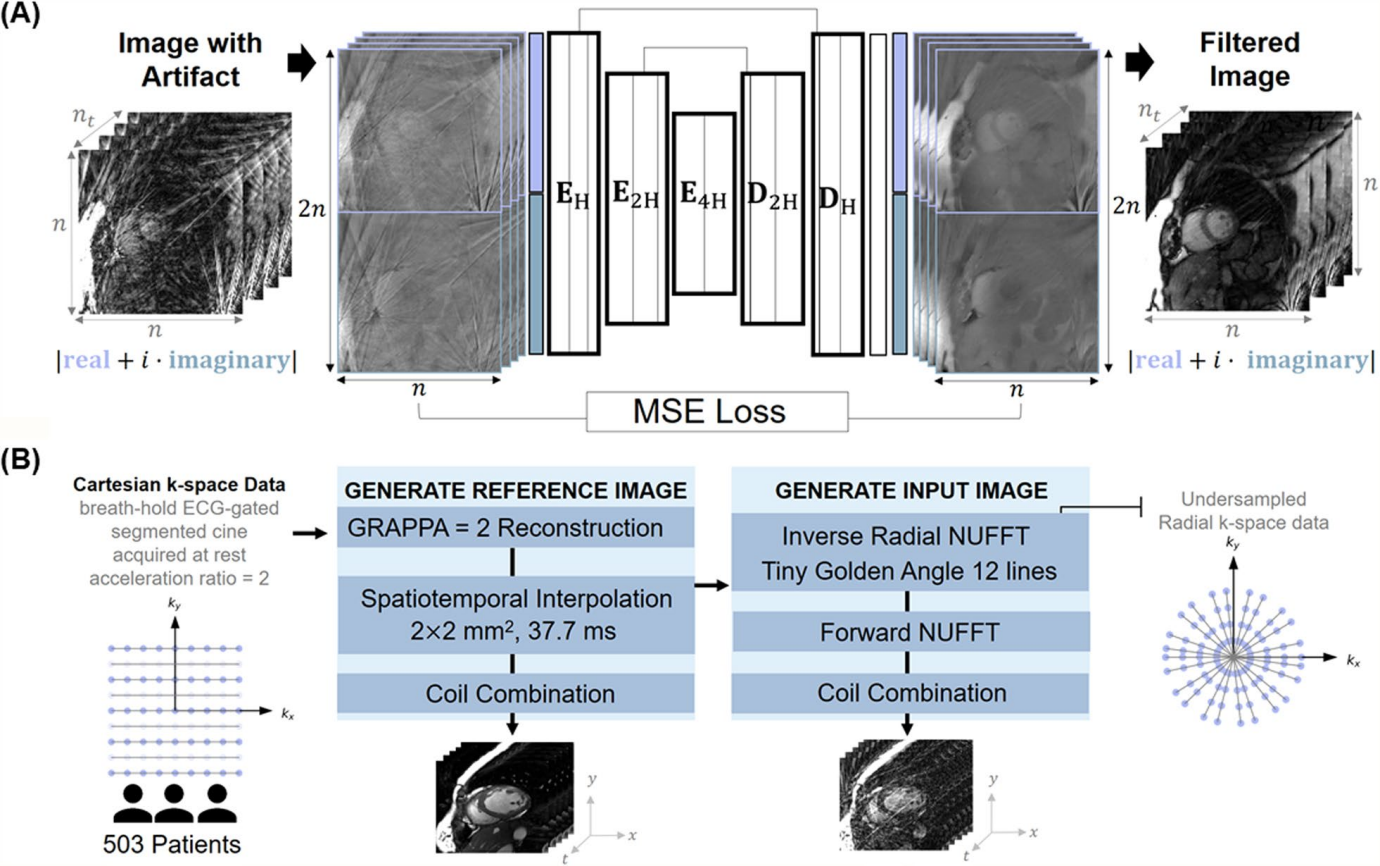
Deep learning model and generation of training data. **A** The deep learning model consists of a three-dimensional U-Net trained to filter out streaking artifacts from complex-valued real-time radial cine $n\times n$ images with ${n}_{t}$ time frames. The inputs and outputs to the U-Net are the real and imaginary components concatenated along the spatial dimension, and the loss during training was the mean square error (MSE) between inputs and outputs. During inference, the generated real and imaginary parts with suppressed artifacts are re-combined. **B** Synthetic real-time image training pairs were generated from electrocardiogram (ECG)-gated segmented Cartesian k-space data acquired at rest in 503 patients. First, the k-space data were reconstructed using the generalized autocalibrating partial parallel acquisition (GRAPPA) technique. Spatiotemporal interpolation followed by coil combination was then applied to the reconstructed images to simulate reference (i.e., ground-truth) real-time images. In addition, prior to coil combination, the interpolated images were also used to simulate undersampled real-time radial cine images by applying an inverse and forward non-uniform fast Fourier transform (NUFFT) with 12 radial lines. The coil-combined images with streaking artifacts were used as inputs during training

The inputs and outputs of the U-Net were single-channel images of size $2n$×$n$×${n}_{t}$, which consisted of concatenated real and imaginary parts of a complex-valued image of size $n$×$n$ with ${n}_{t}$ time frames. C_H_ was defined as a sequence of convolution, batch normalization, drop-out, and rectified linear activation unit layers with H output channels. All convolutions had 3 × 3 × 3 kernel size with 1 × 1 × 1 stride, 1 × 1 × 1 padding, and included bias and dropout level set to 0.15. An encoding layer E_H_ was defined as two sequential C_H_ layers followed by a maximum pooling layer with 2 × 2 × 2 kernel size and 2 × 2 × 2 stride. An encoder was defined as E_H_-E_2H_-E_4H_, where the last encoding layer E_4H_ was without maxpool. A decoding layer D_H_ contained a residual connection, i.e., a transposed convolution with H output channels that was channel-concatenated with the output of E_H_ prior to maxpool, which was followed by two sequential C_H_ layers. All transposed convolutions had 2 × 2 × 2 kernel size with 2 × 2 × 2 stride, zero padding, and included bias. The decoder was defined as D_2H_-D_H.,_ and a final convolution C_f_ with 1 output channel, 1 × 1 × 1 kernel size, 1 × 1 × 1 stride, zero padding, and bias. The U-Net architecture was defined as E_H_-E_2H_-E_4H_-D_2H_-D_H_-C_f_ and is summarized in Fig. 1a.

#### Training data

Cine data from 503 patients at rest (286 males, 55.4 ± 15.8 years) who underwent clinical scans from October 2018 to May 2020 were retrospectively collected. The study cohort consisted of patients with typical clinical indications for CMR, including myocardial scar assessment, non-ischemic cardiomyopathy, and valvular disease. Imaging in these patients was previously performed on a 3 T CMR scanner (MAGNETOM Vida, Siemens Healthineers, Erlangen, Germany). Short axis cine images covering the LV were collected using an ECG-gated segmented sequence with the following parameters: balanced steady-state free-precession (bSSFP) readout, field-of-view (FOV) = 355 × 370 mm^2^, in-plane resolution = 1.7 × 1.4 mm^2^, slice thickness = 8 mm, echo time (TE)/repetition time (TR) = 1.41/3.12 ms, flip angle = 42°, GeneRalized Autocalibrating Partial Parallel Imaging (GRAPPA) acceleration rate = 2, Cartesian sampling pattern, and retrospective ECG-triggering with 25 cardiac phases calculated. To simulate the reference real-time images, the k-space data were first GRAPPA-reconstructed. The images were then resampled to 2 × 2 mm^2^ resolution and 37.7 ms temporal resolution. The ground-truth images for the U-Net were generated using sensitivity-encoding coil combination. Additionally, a backward and forward non-uniform fast Fourier transform (NUFFT) was applied prior to coil combination, which consisted of a tiny golden angle (32.049°) radial trajectory with 12 lines per frame. These undersampled images were coil-combined and used as inputs to the U-Net (Fig. 1b). All steps to generate training data were implemented in MATLAB (MathWorks, Natick, Massachusetts, USA) and are summarized in Fig. 1b.

#### Training

Ground-truth and input images were center-cropped to 144 × 144 and 20 frames. The starting frame was randomly selected to achieve 20 consecutive frames if ${n}_{t}$≥ 20, and the dynamic series was padded to 20 if ${n}_{t}$< 20. During a single training iteration the model was optimized using 16 slices from different patients (i.e., batch size), and for each patient a single slice was randomly selected from the complete short axis stack. Thus, each iteration consisted of ground-truth and input arrays of size 16 × 144 × 144 × 20, which were normalized by the 95^th^ percentile magnitude pixel intensity within the 48 × 48 central region across frames. The U-Net was implemented in PyTorch v1.9.0 (Facebook, Menlo Park, California, USA) and trained for 2,900 iterations. The initial learning rate was set at 0.001 and was reduced by 5% every 100 iterations, using the mean square error loss function and Adam optimizer [29].

#### Inline implementation

**Fig. 2:**
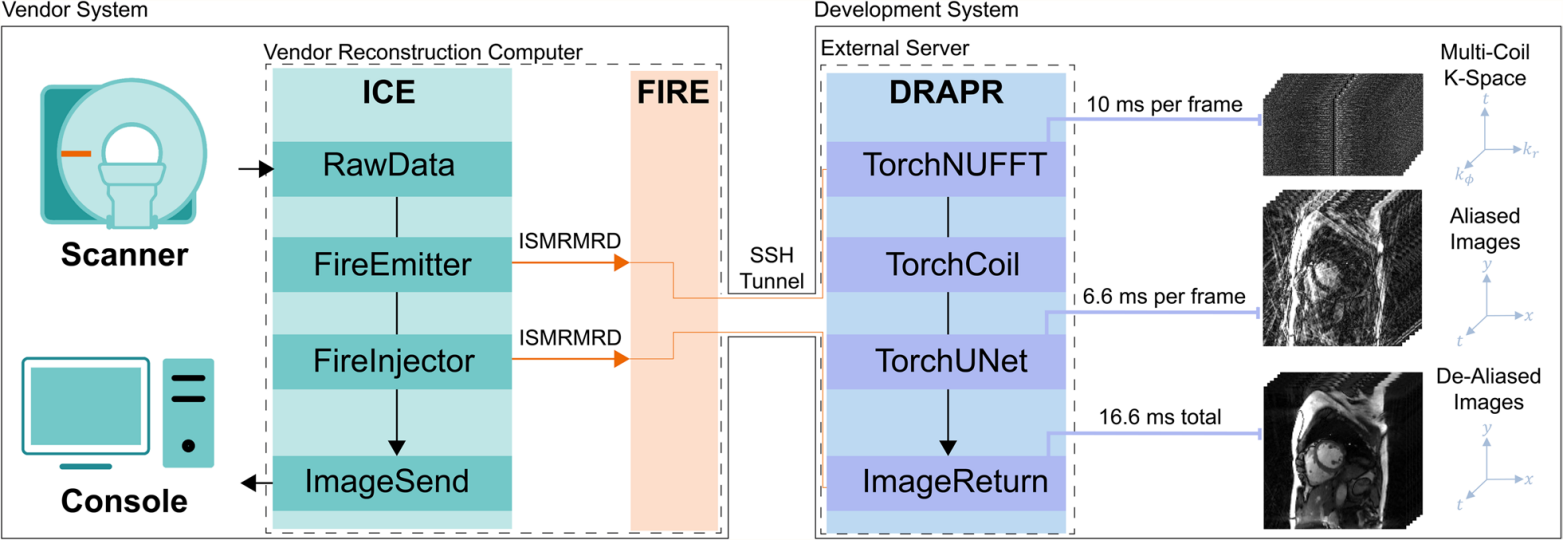
Inline implementation of real-time cine with deep learning-based radial acceleration. Multi-coil raw radial k-space data acquired from the scanner is processed in the Image Reconstruction Environment (ICE) on the vendor reconstruction computer. Using the International Society for Magnetic Resonance in Medicine Raw Data (ISMRMRD) format, the collected data is transferred to the Framework for Image Reconstruction (FIRE) server using a FireEmitter functor. The FIRE server is located in the vendor reconstruction computer. The data is then transferred from the FIRE server to an external server via a connecting Secure Shell Protocol (SSH) tunnel. In the external sever, a Docker containing all Python dependencies such as PyTorch is used to process the raw k-space data in a single 32 GB Graphics Processing Unit (GPU). The deep-learning radial acceleration with parallel reconstruction (DRAPR) technique was implemented in the Docker. First, a non-uniform fast Fourier transform (NUFFT) is used to grid and reconstruct undersampled multi-coil radial k-space data. GPU parallelization is done in PyTorch by treating frames and coils as batch and channel dimensions. This approach enables application of the NUFFT at 10 ms per frame. Coil sensitivity and combination is subsequently performed in PyTorch at negligible computational cost. These coil-combined images are send to the U-Net for de-aliasing, which requires 6.6 ms per frame. The total processing time of 16.6 ms is about half the 37.7 ms temporal resolution of collected frames. Images are then returned to the FIRE server via the same SSH tunnel, and to ICE using a FireInjector functor. Finally, the reconstructed de-aliased images are finalized into DICOM format and returned to the scanner computer console for immediate display

The inline integration was implemented using the Siemens Framework for Image Reconstruction (FIRE) prototype and an external server, which was equipped with 8 Tesla V100 graphics processing units (GPUs), each with 32 GB memory and 5120 cores. All data were processed in the external server. FIRE provided a data streaming interface between the Siemens Image Reconstruction Environment (ICE) in the scanner and the external server environment. Specifically, acquired k-space data was converted to the International Society for Magnetic Resonance in Medicine Raw Data (ISMRMRD) format [30] in real-time and sent to the FIRE server via the FIRE emitter. A secure shell protocol (SSH) tunnel between the vendor reconstruction computer and the external FIRE server provided securely encrypted data streaming. The DRAPR program was deployed in the external server inside a Docker container v20.10.7. Within the Docker, a PyTorch-based NUFFT reconstruction was implemented to enable fast inline reconstruction using a single 32 GB GPU by parallelizing the execution of multiple k-space trajectories. Radial k-space data were reconstructed by executing the torchkbnufft v1.2.0 module [31] with low point precession, 2-neighbor interpolation, and by treating frames and coils as batch and channel dimensions. The coil sensitivity was estimated from the time-averaged NUFFT images using an adaptive coil combination method [32] implemented without smoothing and with a single iteration. Reconstructed images were returned using the FIRE receiver (Fig. 2).

#### Compressed sensing reconstruction

For comparison, the GRASP framework [22] was implemented offline. The following parameters were used: temporal total variation regularization weight = 0.02 [21], line-search operations per iteration = 150 [22], and total number of iterations = 30. Image reconstruction was performed in the external server using a single 32 GB GPU.

### Evaluation

Imaging was prospectively performed on a 3 T CMR scanner (MAGNETOM Vida Siemens Healthineers) using an 18-channel cardiac coil and a 12-channel spine array.

Performance of the proposed approach was evaluated by initially recruiting 8 healthy subjects (3 male, 23 ± 2 years) in whom only rest images were collected to establish the performance of inline DL-accelerated radial imaging at rest. Subsequently, 14 subjects (5 male, 44 ± 21 years) were recruited for an EX-CMR imaging protocol in which both rest and post-exercise images were collected. This cohort consisted of 6 healthy subjects and 8 patients whose inclusion criteria were symptoms of cardiac disease and a previous stress test for suspicion of CAD.

**Fig. 3:**
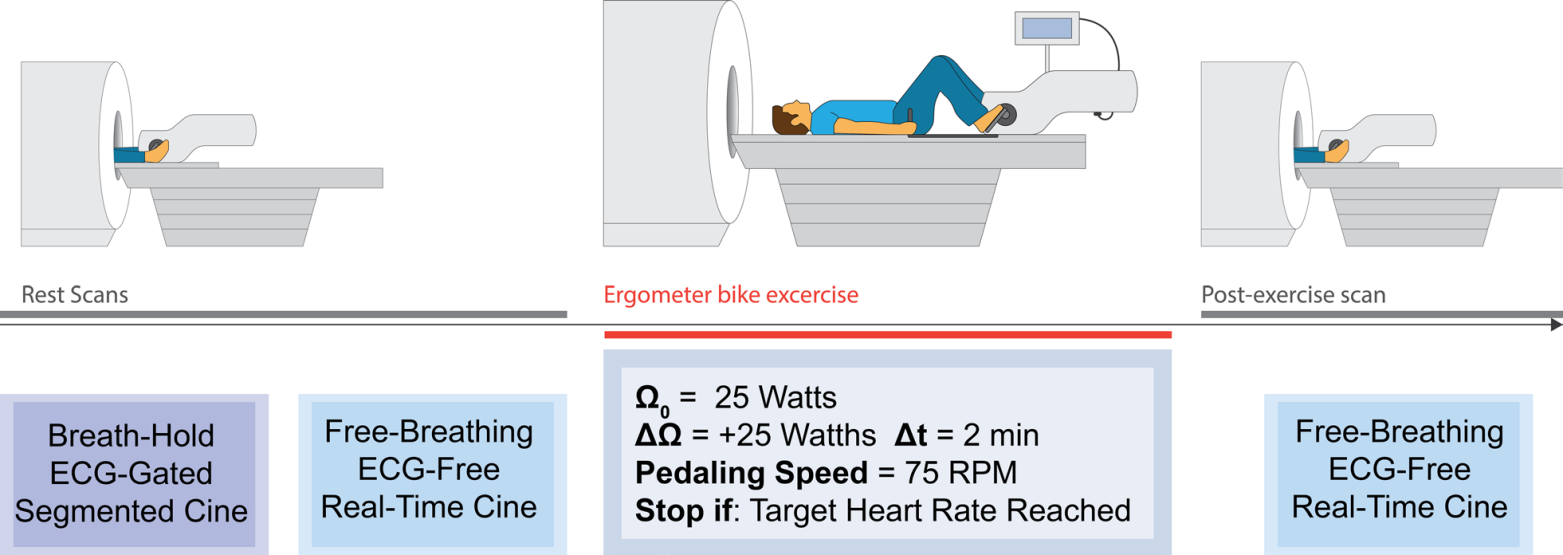
Exercise imaging protocol. Rest scans consisted of breath-hold, ECG-gated Cartesian segmented cine followed by free-breathing ECG-free real-time radial cine. After rest imaging, subjects were removed from the scanner bore and were exercised in the supine position using a cycle ergometer. Work rate was started at 25 W and was increased by + 25 W every 2 min while subjects maintained a constant pedaling speed of 75 rpm. After reaching target heart rate or exhaustion, subjects were immediately placed back inside the scanner bore for post-exercise stress imaging. This consisted of a repetition of the real-time cine sequence

In all subjects, rest imaging consisted of standard breath-hold, ECG-gated Cartesian segmented cine images covering the LV in short axis view collected with the following imaging parameters: bSSFP readout, FOV = 360 × 360 mm^2^, matrix size = 208 × 208, resolution = 1.7 × 1.7 mm^2^, slice thickness = 8 mm, distance factor = 25%, TE/TR = 1.41/3.12 ms, flip angle = 35°, temporal resolution = 53 ms, Cartesian trajectory with GRAPPA acceleration rate = 2, and retrospective ECG-triggering with 25 cardiac phases calculated. In addition, 8 short axis slices of free-breathing ECG-free real-time radial cine images covering the LV were obtained using the DL-accelerated radial imaging prototype with the following parameters: bSSFP readout, FOV = 288 × 288 mm^2^, resolution = 2 × 2 mm^2^, slice thickness = 8 mm, distance factor = 35%, TE/TR = 1.5/3.1 ms, flip angle = 28°, radial lines per phase = 12, tiny golden angle, and temporal resolution = 37.7 ms. In subjects who participated in the Ex-CMR protocol, real-time radial imaging was repeated after exercise using identical parameters. Exercise on these subjects was done using a CMR-compatible cycle ergometer (Lode, Groningen, The Netherlands) outside of the scanner bore after rest imaging. Work rate started at 25 W, and was increased by 25 W every two minutes while maintaining a constant pedaling speed of 75 rpm. Immediately after reaching target heart rate ([220-age] × 0.85) or exhaustion, subjects were returned to the scanner bore for post-exercise stress imaging. Exercise typically lasted 6–12 min, and there was a 4–8 s gap between the end of exercise and stress imaging (Fig. 3).

#### Image analysis

We used both quantitative assessment of LV function and structure and subjective image quality assessment to evaluate the performance of inline DL-accelerated radial real-time cine imaging.

Endocardial and epicardial LV myocardial borders from cine datasets acquired at rest were automatically delineated using an open-source segmentation model [33] and were verified by another reader (M.A.M.). LV end-diastolic and end-systolic volumes (LVEDV and LVESV, respectively) and LV ejection fraction (LVEF) were calculated from the extracted contours.

Three readers (C.T, J.M, and E.S) with > 5 years of experience in CMR were blinded to the acquisition and reconstruction methods, and independently assessed the cine datasets. Datasets were graded on a 4-point Likert scale for level of residual artifacts in the entire FOV (1-non-diagnostic, 2-severe, 3-moderate, 4-minimal).

Statistical analyses were performed using the statistical package for social sciences (SPSS; version 28.0.0.1, Statistical Package for the Social Sciences, International Business Machines, Inc., Armonk, New York, USA). Quantitative measures of LV function were expressed as mean ± standard deviation. Differences in measures of LV function between techniques were evaluated using one-way repeated measures analysis of variance (ANOVA) with post hoc testing using Bonferroni correction. Differences were also visualized using Bland–Altman plots and expressed as mean difference [mean difference ± 1.96 × standard deviation of the difference]. Subjective measures of artifact level scores were expressed as mean ± standard deviation and percentage of cases in each category. Differences in artifact level scores between techniques were evaluated using the Friedman test with Bonferroni correction for images acquired at rest, and the Wilcoxon signed-rank test for images acquired post-exercise. A P-value < 0.05 was considered statistically significant.

## Results

Resting ECG in all patients showed sinus rhythm. Standard cine in one patient showed poor image quality due to off-resonance artifacts. This patient was excluded from the analysis of LV function since proper contouring was not possible, but was included in the analyses of image quality. In addition, one of the patients was unable to complete the Ex-CMR protocol due to a leg cramp during supine cycle exercise. The baseline and post-exercise heart rates were 72 ± 15 and 124 ± 19 bpm, which represent an average increase of 53 ± 20 bpm.

Reconstruction of real-time radial cine images with GRASP and DRAPR was successful in all cases. Using GRASP to reconstruct cine data whose frame matrix size was 288 × 288 required 1.2 s per frame for pre-processing and 6.1 s per frame for application of compressed sensing to the coil-combine complex images. With DRAPR, pre-processing times of 10 ms per frame were achieved, and application of the U-Net model to the coil-combine complex images was achieved in 6.6 ms per frame. Thus, for instance, an acquisition over 4 heart beats (i.e., ~ 100 frames) and 12 slices would require 2.4 h with GRASP and 20 s with DRAPR.

**Fig. 4:**
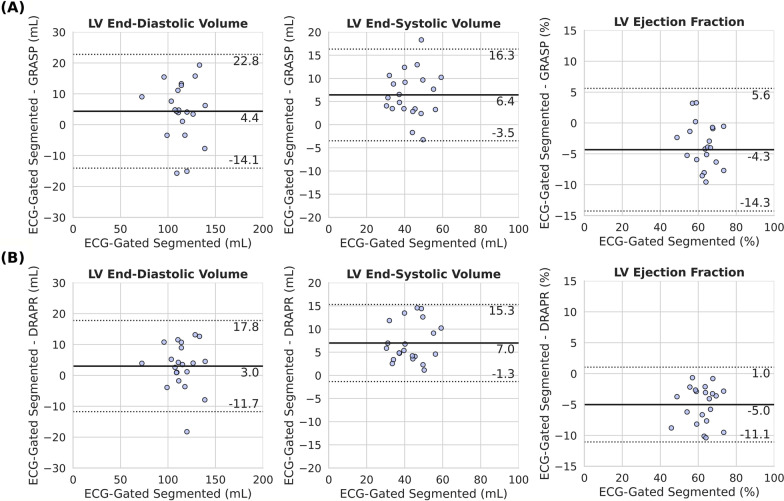
Bland–Altman comparisons of left-ventricular (LV) parameters. Measures derived from breath-hold ECG-gated segmented cine are compared to those derived from the free-breathing ECG-free real-time cine reconstructed with the GRASP and deep-learning radial acceleration with parallel reconstruction (DRAPR) techniques. Solid and dotted lines represent the mean difference and mean difference ± 1.96 × standard deviation of the difference. **A** The mean differences in LV end-diastolic and end-systolic volumes with golden angle radial sparse parallel (GRASP) were 4.4 mL and 6.4 mL, while the mean difference in LV ejection fraction was − 4.3%. **B** The mean differences in LV end-diastolic and LV end-systolic volumes with DRAPR were 3 mL and 7 mL, while the mean difference in ejection fraction was − 5%

Measures of LV function across all subjects based on breath-hold ECG-gated segmented cine and free-breathing ECG-free real-time radial cine reconstructed with GRASP and DRAPR were 114 ± 15 mL, 118 ± 17 mL, and 117 ± 17 mL for LVEDV; 43 ± 9 mL, 49 ± 10 mL, 50 ± 10 mL for LVESV; and 62 ± 7%, 58 ± 10%, 57 ± 7% for LVEF. Comparison of measures based on ECG-gated and GRASP cine showed differences in LVEDV (4.4 mL [− 14.1, 22.8], P = 0.138) that were not significantly different from zero. However, differences in LVESV (6.4 mL [− 3.5, 16.3], P < 0.001) and LVEF (− 4.3% [− 14.3, 5.6], P = 0.003) were significant. Similarly, comparison of measures based on ECG-gated and DRAPR showed differences in LVEDV (3.0 mL [− 11.7, 17.8], P = 0.320) that were not significantly different from zero. Differences in LVESV (7.0 mL [− 1.3, 15.3], P < 0.001) and LVEF (− 5.0% [− 11.1, 1.0], P < 0.001) were significant. There were no significant differences between GRASP- and DRAPR-based measures of LV function (Fig. 4).

**Fig. 5:**
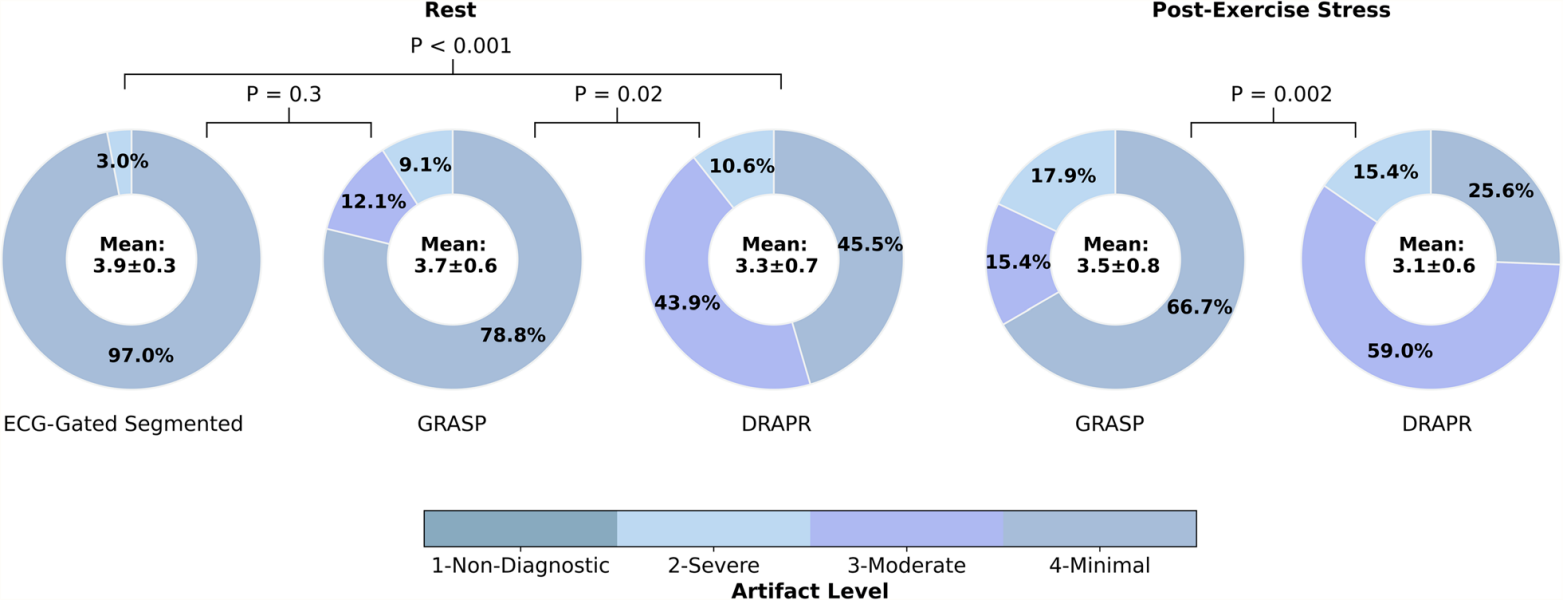
Subjective evaluation of image quality. Three readers evaluated artifacts across all cine scans. Images were classified as having a 1-non-diagnostic, 2-severe, 3-moderate, and 4-minimal artifact level. The mean score of breath-hold ECG-gated segmented cine images was 3.0. The mean score of free-breathing ECG-free real-time cine images reconstructed with GRASP was 3.7 at rest, with 9.1% of images graded as severe. The mean score during post-exercise stress was 3.5, and the percentage of images graded as severe increased to 17.9%. Real-time cine images at rest reconstructed with DRAPR had a mean score of 3.3, with 10.6% of images graded as severe. During post-exercise stress, images had a mean score of 3.1. The percentage of images graded as severe increased to 15.4%. None of the real-time radial cine images reconstructed with GRASP or DRAPR were labeled as non-diagnostic. *p < 0.01. GRASP: golden-angle radial sparse parallel; DRAPR: deep-learning radial acceleration with parallel reconstruction

**Fig. 6:**
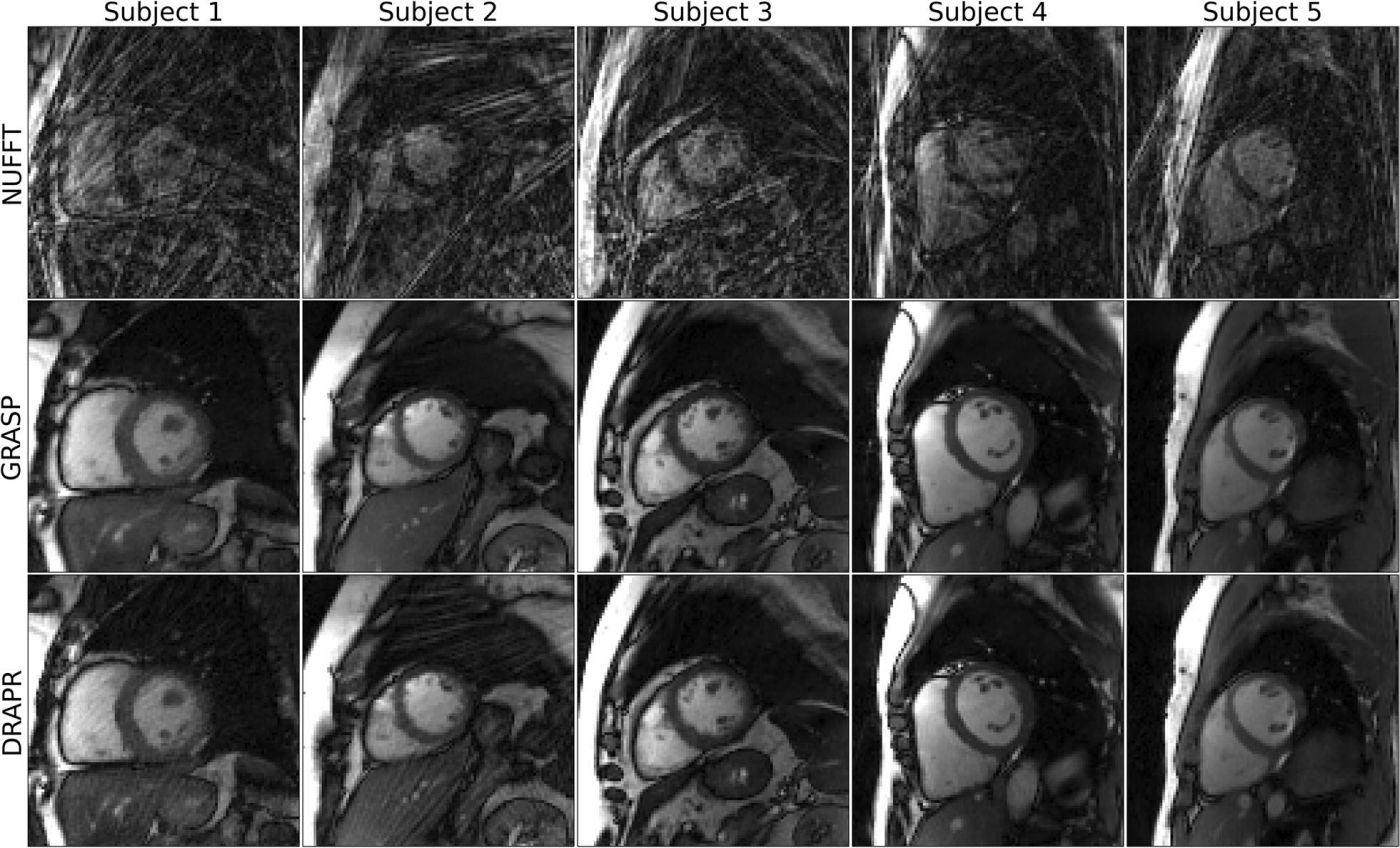
Representative real-time cine images of subjects at rest. The NUFFT was used to grid and reconstruct radial k-space data. Artifacts were subsequently suppressed using the GRASP and DRAPR techniques. Images were classified as having a 1-non-diagnostic, 2-severe, 3-moderate, and 4-minimal artifact level. All NUFFT images were 1-non-diagnostic. The five subjects shown at end-diastole represent the range of mean scores at rest. Subjects 1–4 are patients. The mean artifact levels with DRAPR for subjects 1–5 were 3.0, 3.3, 3.3, 4.0 and 4.0, accordingly. The body mass index (BMI) for subjects 1–5 was 38, 33, 26, 23 and 21 lbs/in^2^, accordingly

**Fig. 7:**
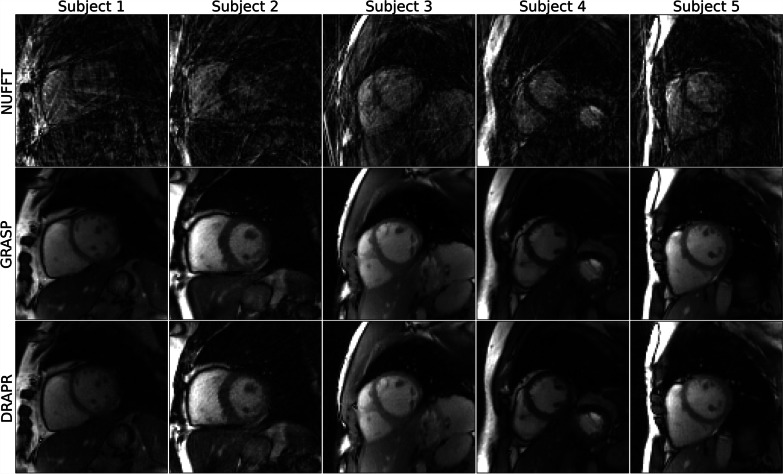
Representative real-time cine images of subjects at post-exercise stress. The NUFFT was used to grid and reconstruct radial k-space data. Artifacts were subsequently suppressed using the GRASP and DRAPR techniques. Images were classified as having a 1-non-diagnostic, 2-severe, 3-moderate, and 4-minimal artifact level. All NUFFT images were 1-non-diagnostic. The five subjects shown at end-diastole represent the range of mean scores at post-exercise stress. Subjects 3–4 are healthy. Subjects 2 and 5 correspond to Subjects 1 and 4 in Fig. 6, accordingly. The mean artifact levels with DRAPR for subjects 1–5 were 2.3, 3.0, 3.0, 3.3 and 3.7, accordingly. The body mass index (BMI) for subjects 1–5 was 32, 38, 23, 29 and 24 lbs/in^2^, accordingly. The peak heart rate post-exercise for subjects 1–5 was 110, 108, 97, 132, and 135 bpm, accordingly

The mean artifact level score in ECG-gated segmented cine images was 3.9 ± 0.3. Only 3.0% of the scores were graded as severe and the remaining 97.0% were graded as minimal. All NUFFT-reconstructed real-time cine images were deemed as having a non-diagnostic level of artifacts, while none remained non-diagnostic after application of GRASP or DRAPR. The mean score in real-time cine images at rest reconstructed with GRASP was 3.7 ± 0.6, which was comparable (P = 0.3) to ECG-gated images. However, 9.1% of scores were graded as severe. The mean scores of images reconstructed with DRAPR was 3.3 ± 0.7, which reflects a significantly higher artifacts relative to ECG-gated (P < 0.001) and GRASP (P = 0.02) images. In addition, 10.6% of scores were graded as severe (Fig. 5). Various subjects representing a range of scores are shown in Fig. 6 and Additional file 1: Video 1, i.e., from a mean score of 3 (reader A, B, C = 2, 4, 3) to a mean score of 4 (reader A, B, C = 4, 4, 4) with DRAPR. The artifact level of images reconstructed with GRASP (3.5 ± 0.8) and DRAPR (3.1 ± 0.6) during post-exercise stress was significantly different (P = 0.002). Relative to scores of images at rest, the percentage of scores graded as severe increased to 17.9% and 15.4%, accordingly. A range from a mean artifact score of 2.3 with DRAPR (reader A, B, C = 2, 3, 2) to 3.7 (reader A, B, C = 3, 4, 4) in subjects post-exercise is shown in Fig. 7 and Additional file 2: Video 2.

**Fig. 8:**
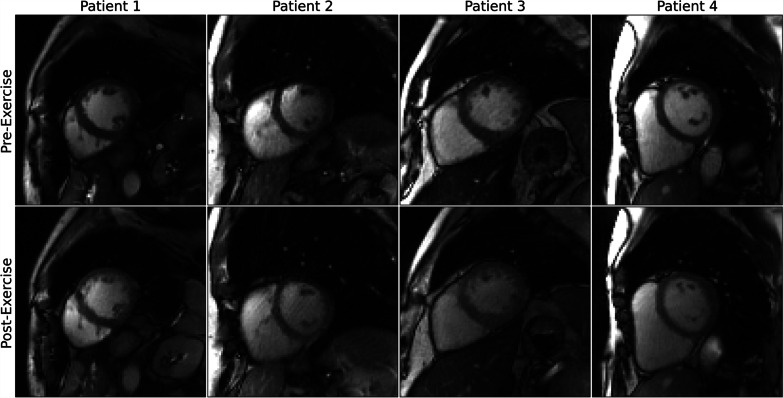
Inline real-time cine with deep learning-based radial acceleration in coronary artery disease (CAD). Four patients are shown whose recruitment criteria included being symptomatic and having a stress test for suspicion of CAD. Patients underwent an exercise CMR protocol. Top images were collected at rest, and bottom images were collected after supine exercise using a CMR compatible cycle ergometer. Images are shown at end-diastole. Patient 4 at pre-exercise corresponds to subject 4 in Fig. 6, and at post-exercise corresponds to subject 5 in Fig. 7, accordingly. The BMI for patients 1–4 was 22, 27, 26, and 24 lbs/in^2^, accordingly. During rest, the mean artifact levels were 3.0, 3.7, 4.0 and 4.0. The resting heart rates were 83, 62, 70 and 49 bpm. During post-exercise stress, the mean artifact levels were 3.3, 3.3, 3.3 and 3.7. The peak heart rates post-exercise were 115, 132, 107 and 134 bpm, accordingly

**Fig. 9:**
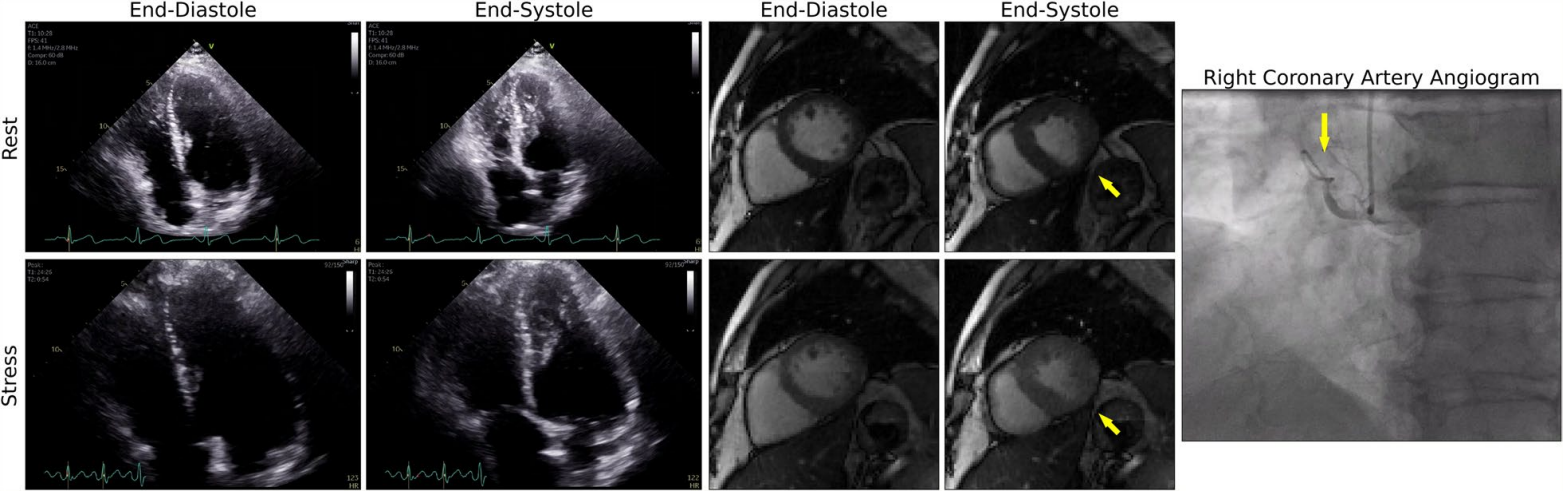
Illustrative clinical case. A 60-year-old male with worsening upper chest heaviness and shortness of breath with exertion. Stress echo showed focal systolic dysfunction with hypokinesis of the left-ventricular basal inferoseptum and inferior walls at both rest and stress. Similarly, during an exercise CMR protocol, real-time cine with deep learning-based radial acceleration showed hypokinesis of the left-ventricular basal inferoseptal wall during rest and at post-exercise stress (arrows). Subsequent coronary angiography revealed a heavily calcified right coronary artery (arrow) with serial subtotal occlusions

Representative real-time cine images reconstructed with DRAPR in patients with suspected CAD are shown in Fig. 8 and Additional file 3: Video 3. Ex-CMR showed absence of regional wall motion abnormalities in seven of eight patients, which was consistent with findings from other modalities. Specifically, exercise tolerance test (ETT) was negative in two patients. Stress echocardiography tests were also negative in three patients, and nuclear stress showed a moderate perfusion defect in one patient. No other imaging tests were performed in any of these six patients. In one patient, stress echocardiography was positive but subsequent coronary angiography was negative for significant CAD. In the remaining patient whose Ex-CMR showed a regional wall motion abnormality, stress echocardiography showed hypokinesis of the LV basal inferoseptum, and coronary angiography revealed a heavily calcified right coronary artery with subtotal and chronic total occlusions (Fig. 9 and Additional file 4: Video 4).

## Discussion

In this study, we assessed the feasibility of inline real-time cine with DL-based radial acceleration for Ex-CMR. We demonstrated that (1) while our training dataset was based on synthetic real-time images simulated from images acquired during breath-hold and at rest, DRAPR substantially suppressed artifacts in free-breathing real-time cine images acquired during post-exercise stress; (2) the proposed inline implementation for seamless, low-latency reconstruction is a feasible approach for evaluation of cardiac function in Ex-CMR.

Ex-CMR with real-time radial cine has the potential to detect regional wall motion abnormalities in patients with suspected CAD. Fractional flow reserve (FFR) is the gold standard invasive diagnostic test to assess functional significance of coronary artery stenosis [34]. However, studies suggest that CMR can provide highly sensitive and specific CAD assessment compared to invasive FFR, and may be non-inferior at predicting adverse events [4, 35, 36]. Compared to pharmacological stress, Ex-CMR may provide additional exercise-capacity and hemodynamic prognostic information and elicit a more powerful stress response. Thus, exercise stress is preferable for cardiac stress testing when feasible. Despite its clinical importance, the clinical usefulness of Ex-CMR has been limited by lack of adequate temporal resolution and image quality due to elevated heart rates, exaggerated breathing, and gross patient motion during imaging. In recent years, the advent of highly accelerated sequences have enabled free-breathing ECG-free cine imaging [13]. In this feasibility study, we implemented a prototype cine sequence with DL-based radial acceleration and spatiotemporal resolution of 37.7 ms and 2 × 2 mm^2^, respectively. Our inline implementation was able to quickly reconstruct and suppress streaking artifacts in 16.6 ms per frame, and evaluation of LV function in all patients with suspected CAD using both rest and post-exercise cine images was in agreement with other stress imaging modalities.

Isometric handgrip was the first exercise method proposed for Ex-CMR [14]. Supine ergometry was later proposed by Mohiaddin et al. as an alternative exercise method that does not require patient repositioning and engages large muscle groups [37]. Previous Ex-CMR studies have reported exercise can be performed inside the scanner bore using a cycle ergometer [20]. However, we found that many patients are not able to adequately cycle inside the scanner due to limitations of the bore size. With our approach, there was only a small delay from peak exercise intensity to stress imaging. In addition, with the exception of one patient who experienced a leg cramp during supine cycling, all healthy subjects and patients were able to complete the Ex-CMR protocol.

The underestimation of LVEF in real-time cine relative to ECG-gated was due to greater volume overestimation at end-systole compared to end-diastole. Physiologic and parameter variations between ECG-gated and real-time imaging could lead to systematic differences in LV parameters. Further, our three-dimensional U-Net implementation applies a temporal filtering that could result in reduced temporal resolution and may explain the LVEF underestimation. In addition, it might also make visual detection of regional wall motion abnormalities more difficult, especially during post-exercise or dobutamine stress. Alternatively, a recurrent two-dimensional U-Net could avoid temporal smoothing. Nevertheless, these volume differences in volume were smaller than those reported in a previous validation study of a similar real-time radial cine sequence reconstructed with GRASP [21]. In that study, comparison to ECG-gated quantification of LV parameters showed mean differences in LVEDV and LVESV of 15.2 mL and 7.9 mL, respectively.

In this study, we also compared DRAPR- to GRASP-based dealiasing. Although the artifact level scores were higher with GRASP, the difference between the two was small and may not necessarily impact clinical interpretation or quantification. Indeed, we found no significant differences between GRASP- and DRAPR-based measures of LV function, and both methods received a similar amount of severe artifact scores. Further, DRAPR was able to de-aliased images with a 1000-fold reduction in computational time compared to GRASP. In Ex-CMR studies, cine images may be acquired over multiple heart beats and multiple slices. DRAPR enables inline reconstruction of these images in the order of seconds instead of hours, which is important to prevent delays in the clinical workflow.

We did not observe any dependence on heart rate in the artifact scores in our study. However, subjects with high body mass index showed increased artifact levels. The lowest artifact level scores at rest represent reduced suppression of streaking artifacts arising from high-intensity signals and field inhomogeneities in the peripheral field-of-view. Since the peripheral FOV normally contains a higher amount of artifacts, better artifact suppression could also be achieved by using the entire image matrix during training. However, such implementation would require larger GPU memory. Alternatively, a patch-level training approach could perform better than or similar to an image-level approach with reduced memory demand.

Low latency enables quick evaluation of images, and also prevents computational bottlenecks that may occur in imaging protocols with finite computing resources. Our integration enabled us to perform NUFFT reconstruction and coil combination in 10 ms per frame, which was almost one quarter of the temporal resolution. This approach could be used to speed up previously proposed DL methods. For example, in the model proposed by Shen et al. for real-time radial cine, these pre-processing steps were done in ~ 0.3 s per frame [27]. Better artifact suppression might be possible if additional GPUs are available, or in protocols where some latency can be sacrificed. For instance, we concatenated real and imaginary components to enable DL processing with real-valued convolutional kernels. Alternatively, complex-valued convolutions may be used, but would require a two-fold increase in the number of parameters and a four-fold increase in the number of operations. Also, the artifact level in gridded images could be reduced by increasing the number of neighbors and point precision in the NUFFT, or by including prior smoothing and increasing the number of iterations during coil sensitivity estimation. Nevertheless, we showed that streaking artifacts were substantially suppressed using our fast inline model in various Ex-CMR studies in both healthy subjects and patients with suspected CAD.

### Limitations

The present report represents a proof-of-concept study with a small sample size. Only one patient had a confirmed regional wall motion abnormality associated with a coronary occlusion. Therefore, our study was too small to assess diagnostic accuracy. Our quantification of LV measures was limited since we used breath-hold, ECG-gated segmented cine images to obtain reference values. Also, real-time cine images were acquired at only 8 slices with a 35% distance gap, which could cause a systematic bias in measures of LV volumes. We also did not evaluate the optimal choice of DL architecture; however, the proposed implementation showed excellent artifact suppression with minimal reconstruction latency. Finally, data from a single vendor and field strength were used for training, and the generalizability of the trained network should be studied.

## Conclusion

Combination of highly accelerated radial sequences with DL enables fast acquisition and reconstruction of real-time cine images with suppressed streaking artifacts. Our study demonstrated the feasibility of low-latency, inline implementation of such methodology for clinical Ex- CMR.

## Supplementary Information

**Additional file 1**. Representative real-time cine videos of subjects at rest. The non-uniform fast Fourier transform (NUFFT) was used to grid and reconstruct radial k-space data. Artifacts were subsequently suppressed using the GRASP and DRAPR techniques, as shown by the sliding window in the videos. Images were classified as having a 1-non-diagnostic, 2-severe, 3-moderate, and 4-minimal artifact level. All NUFFT images were 1-non-diagnostic. The five subjects shown at end-diastole represent the range of mean scores at rest. Subjects 1–4 are patients. The mean artifact levels with DRAPR for subjects 1–5 were 3.0, 3.3, 3.3, 4.0 and 4.0, accordingly. The body mass index (BMI) for subjects 1–5 was 38, 33, 26, 23 and 21 lbs/in2, accordingly. GRASP: golden-angle radial sparse parallel; DRAPR: deep-learning radial acceleration with parallel reconstruction.

**Additional file 2**. Representative real-time cine videos of subjects at post-exercise stress. The non-uniform fast Fourier transform (NUFFT) was used to grid and reconstruct radial k-space data. Artifacts were subsequently suppressed using the GRASP and DRAPR techniques, as shown by the sliding window in the videos. Images were classified as having a 1-non-diagnostic, 2-severe, 3-moderate, and 4-minimal artifact level. All NUFFT images were 1-non-diagnostic. The five subjects shown at end-diastole represent the range of mean scores at post-exercise stress. Subjects 3–4 are healthy. The mean artifact levels with DRAPR for subjects 1–5 were 2.3, 3.0, 3.0, 3.3 and 3.7, accordingly. The BMI for subjects 1–5 was 32, 38, 23, 29 and 24 lbs/in2, accordingly. The peak heart rate post-exercise for subjects 1–5 was 110, 108, 97, 132, and 135 bpm, accordingly. GRASP: golden-angle radial sparse parallel; DRAPR: deep-learning radial acceleration with parallel reconstruction.

**Additional file 3**. Videos of inline real-time cine with deep learning-based radial acceleration in coronary artery disease (CAD). Four patients are shown whose recruitment criteria included being symptomatic and having a stress test for suspicion of coronary artery disease (CAD). Patients underwent an exercise CMR protocol. Top images were collected at rest, and bottom images were collected after supine exercise using a CMR compatible cycle ergometer. Images are shown at end-diastole. The BMI for patients 1–4 was 22, 27, 26, and 24 lbs/in2, accordingly. During rest, the mean artifact levels were 3.0, 3.7, 4.0 and 4.0. The resting heart rates were 83, 62, 70 and 49 bpm. During post-exercise stress, the mean artifact levels were 3.3, 3.3, 3.3 and 3.7. The peak heart rates post-exercise were 115, 132, 107 and 134 bpm, accordingly.

**Additional file 4**. Video of illustrative clinical case. A 60-year-old male with worsening upper chest heaviness and shortness of breath with exertion. Stress echo showed focal systolic dysfunction with hypokinesis of the left-ventricular basal inferoseptum and inferior walls at both rest and stress. Similarly, during an exercise CMR protocol, real-time cine with deep learning-based radial acceleration showed hypokinesis of the left-ventricular basal inferoseptal wall during rest and at post-exercise stress (arrows). Subsequent coronary angiography revealed a heavily calcified right coronary artery (arrow) with serial subtotal occlusions.

## Data Availability

The proposed model is an investigational technique and not available by the vendor as a research tool or product. The model codes are openly available on GitHub: https://github.com/HMS-CardiacMR/DRAPR. The datasets supporting the conclusions of this article are available in the Harvard dataverse: https://dataverse.harvard.edu/dataverse/cardiacmr. Identifying scheme: ORCID number 0000-0003-4025-7399.

## Abbreviations

BMI: Body mass index
bSSFP: Balanced steady-state free-precession
CAD: Coronary artery disease
CMR: Cardiovascular magnetic resonance
DL: Deep learning
DRAPR: Deep-learning radial acceleration with parallel reconstruction
ECG: Electrocardiogram
ETT: Exercise tolerance test
Ex-CMR: Exercise cardiovascular magnetic resonance
FFR: Fractional flow reserve
FIRE: Framework for image reconstruction
FOV: Field-of-view
GRAPPA: Generalized autocalibrating partial parallel imaging
GRASP: Golden angle radial sparse parallel
ICE: Image reconstruction environment
ISMRMRD: International Society for Magnetic Resonance in Medicine Raw Data
LV: Left ventricle/left ventricular
LVEDV: Left-ventricular end-diastolic volume
LVEF: Left-ventricular ejection fraction
LVESV: Left-ventricular end-systolic volume
NUFFT: Non-uniform fast Fourier transform
SSH: Secure shell protocol
TE: Echo time
TR: Repetition time
